# Experimental study on deformation characteristics of seasonal subgrade soil under dynamic load

**DOI:** 10.1371/journal.pone.0309443

**Published:** 2024-08-26

**Authors:** Dongwei Li, Zecheng Wang, Zhiwen Jia, Wenjie Bu, Qiao Sun, Zhenhua Wang

**Affiliations:** 1 College of Civil Engineering and Architecture, Dalian University, Dalian, China; 2 School of Civil and Architectural Engineering, East China University of Technology, Nanchang, China; College of Engineering Guindy, Anna University, Chennai, India, INDIA

## Abstract

In Northwest China, the highway infrastructure often faces challenges due to the widespread presence of subgrade soil. This soil undergoes significant changes in performance under cyclic loading and freeze-thaw cycles. To effectively design and construct highways in these regions, it is crucial to understand the impact of various factors on the deformation characteristics and mechanical properties of subgrade soil. This study aims to investigate the influence of freeze-thaw cycles, water content, confining pressure, and loading rate on the deformation behavior and mechanical properties of subgrade soil under cyclic loading conditions. Experimental tests were conducted to analyze the deformation characteristics and mechanical properties of the subgrade soil. The test results revealed the following: 1) Dynamic loading leads to a noticeable decrease in the strength of subgrade soil, resulting in a softening effect on the stress-strain curve. The cumulative strain of the soil is positively correlated with the number of freeze-thaw cycles and water content, while negatively correlated with confining pressure. The final cumulative strain remains below 1%. 2) The failure stress of subgrade soil decreases exponentially with an increase in freeze-thaw cycles, dropping from 224.52 kPa to 196.76 kPa. 3) An increase in water content linearly decreases the failure stress of subgrade soil, ranging from 377.1 kPa to 151.5 kPa. 4) Confining pressure exhibits a linearly increasing relationship with the failure stress of subgrade soil, ranging from 151.6 kPa to 274.5 kPa. 5) The failure stress of subgrade soil demonstrates a linear increase with the loading rate, ranging from 200.46 kPa to 210.62 kPa. These findings provide valuable insights for the design and construction of highways in seasonal frozen areas. They also offer guidance for preventing and mitigating subgrade freeze-thaw issues in the future.

## 1. Introduction

China has the world’s third largest frozen soil area, including both permafrost and seasonal frozen soil regions in the western and central parts of the country. These areas cover 53.5% of the land [1, 2]. With increased infrastructure investment in recent years, there has been a rise in the construction of railway and highway projects in cold regions. When vehicles or machines apply vibration loads, the soil undergoes dynamic deformation under both dynamic and static stress. This process is not a simple addition, but rather a complex mechanical process [3]. While conventional soil mechanics has made significant progress in studying soil dynamics, the study of frozen soil dynamics, particularly in seasonal frozen soil, is still in its early stages. When dynamic loads act on frozen soil, the soil deformation continues to increase due to the combined effects of freeze-thaw cycles and fatigue from the dynamic load, even if the frequency and amplitude remain constant. Additionally, the internal structure of seasonal frozen soil changes significantly due to repeated fluctuations in internal temperature [4–6]. Scholars such as Zhao Shuping, Zhang Zhimin, and Chen Dun [7–9] have categorized and summarized the main research problems in frozen soil dynamics into three categories: strength, deformation, and stability.

At present, dynamic research is primarily conducted through dynamic load triaxial tests, creep tests, and CT tests [10–12]. Kamei T et al. [13] investigated the influence of freeze–thaw cycles on the unconfined compressive strength and durability of very soft clay soil stabilised with recycled Bassanite, which is produced from gypsum wastes. Study show that an increase in the number of freeze–thaw cycles decreases the unconfined compressive strength and durability index. M Roustaei et al. [14] conducted uniaxial compression and tensile splitting tests to investigate the impact of nano-clay on the mechanical properties of clay. These tests were conducted using various proportions of nano-clay, as well as different curing times and freeze-thaw cycles. Qiu P et al. [15] study the creep evolutions of soil-rock mixture (SRM) under various F-T cycles (0 to 15 cycles) and rock contents (15% to 55%). A new element combination creep model was then proposed to describe SRM’s nonlinear creep behaviors involving the instantaneous elastoplastic and viscoelastic-plastic deformations. Cui et al. [16], by analyzing the results of cyclic triaxial tests, discovered that the dynamic shear modulus of saturated silty sand decreases the most after one freeze-thaw cycle. After 14 freeze-thaw cycles, both the dynamic shear modulus and damping ratio no longer fluctuate. Based on this, a modified Hardin hyperbolic model is established, and an empirical formula for predicting the dynamic shear modulus and damping ratio is proposed.

Lu et al. [17] presented a cumulative deformation model that takes into account the influence of dynamic deviator stress, number of cycles, and material properties of the subgrade soil. They derived and calculated the induced stress of traffic load based on a vehicle-road-ground coupling model. Qi et al. [18] conducted dynamic triaxial tests to study the time evolution and spatial distribution of strain and pore pressure in highway subgrade soil under traffic load. They analyzed the impact of traffic load on subgrade deformation and developed a numerical calculation model. An et al. [19] monitored the load, vehicle speed, and dynamic pressure distribution at different depths under 25 working conditions. They investigated the dynamic pressure stress response of heavy truck movement on pavement subgrade. Wang et al. [20] designed a special loading device and used a 450kV industrial X-ray CT machine to test the deformed subgrade.

There are also many scholars who use model tests, numerical analysis, and theoretical analysis methods [21, 22]. Fu et al. [23] conducted an experimental study on nine groups of frozen soil samples with an initial water content of 20% under different test conditions using a split Hopkinson pressure bar. They established a constitutive model to predict the dynamic strength and compressive deformation of frozen soil under impact load. Cui et al. [24] studied the influence of different axle loads, speeds, and vehicle types on the dynamic response of the subgrade and the spatial distribution of vertical dynamic stress of the highway subgrade through model tests. They also established an estimation model for the influence depth of vertical dynamic stress and vehicle load. Zhang et al. [25] combined field monitoring with indoor model tests to study the dynamic characteristics of the subgrade. They monitored the temperature at different depths for nearly 1 year and conducted dynamic analysis. Sun et al. [26] simulated various working conditions of a composite subgrade through indoor model tests and studied the influence of different factors on the stress distribution and consolidation drainage of the composite foundation. Li et al. [27] proposed a subgrade model test system that can simulate water immersion. They used this system to carry out dynamic response tests on the subgrade under different working conditions.

The freeze-thaw cycle of seasonal frozen soil deteriorates the physical properties of subgrade soil, which affects its bearing capacity. This can lead to various issues such as frost heave, frost boiling, and other phenomena, resulting in road subsidence, bulging, deflection, and other problems that cannot be ignored [28, 29]. Due to the low level of early construction technology, completed highways have experienced long-term traffic operation, causing performance degradation under traffic load and an increased incidence of diseases [30].

As mentioned earlier, previous studies have primarily focused on examining the impact of various factors on the stability and strength of subgrade soil, without taking into account the changes in strength and stability that occur after prolonged exposure to vehicle loads. This paper aims to address this gap by investigating the deformation characteristics of typical silty highway subgrade soil in Northwest China under cyclic loading, as well as its mechanical properties following long-term vehicle load. To achieve this, we will be studying the influence of different factors such as freeze-thaw cycles, water content, confining pressure, and loading rate. The findings of this study will not only contribute to the design and construction of expressways in seasonal frozen regions in the future, but also offer valuable insights into the prevention and treatment of subgrade freeze-thaw diseases.

## 2. Test introduction

In order to accurately represent the soil’s unsaturated state in the actual project in the northwest region of China, we have selected the optimal water content of 10.4% as the intermediate value. The water content of the remolded soil has been set at 6%, 8%, 10%, and 12%. The dry density is 1.93g/cm^3^, achieved through a compaction degree of 96%. To account for the common horizontal stress range of the subgrade, we have chosen three grades: 50 kPa, 100 kPa, and 150 kPa. Additionally, in order to examine the impact of the number of freeze-thaw cycles, we have set the negative temperature for 0, 1, 3, 5, and 7 cycles to a fixed value of -10°C. In this study, the dynamic stress amplitude is 0–0.2 / 0.4 / 0.6 times static strength.

**Fig 1:**
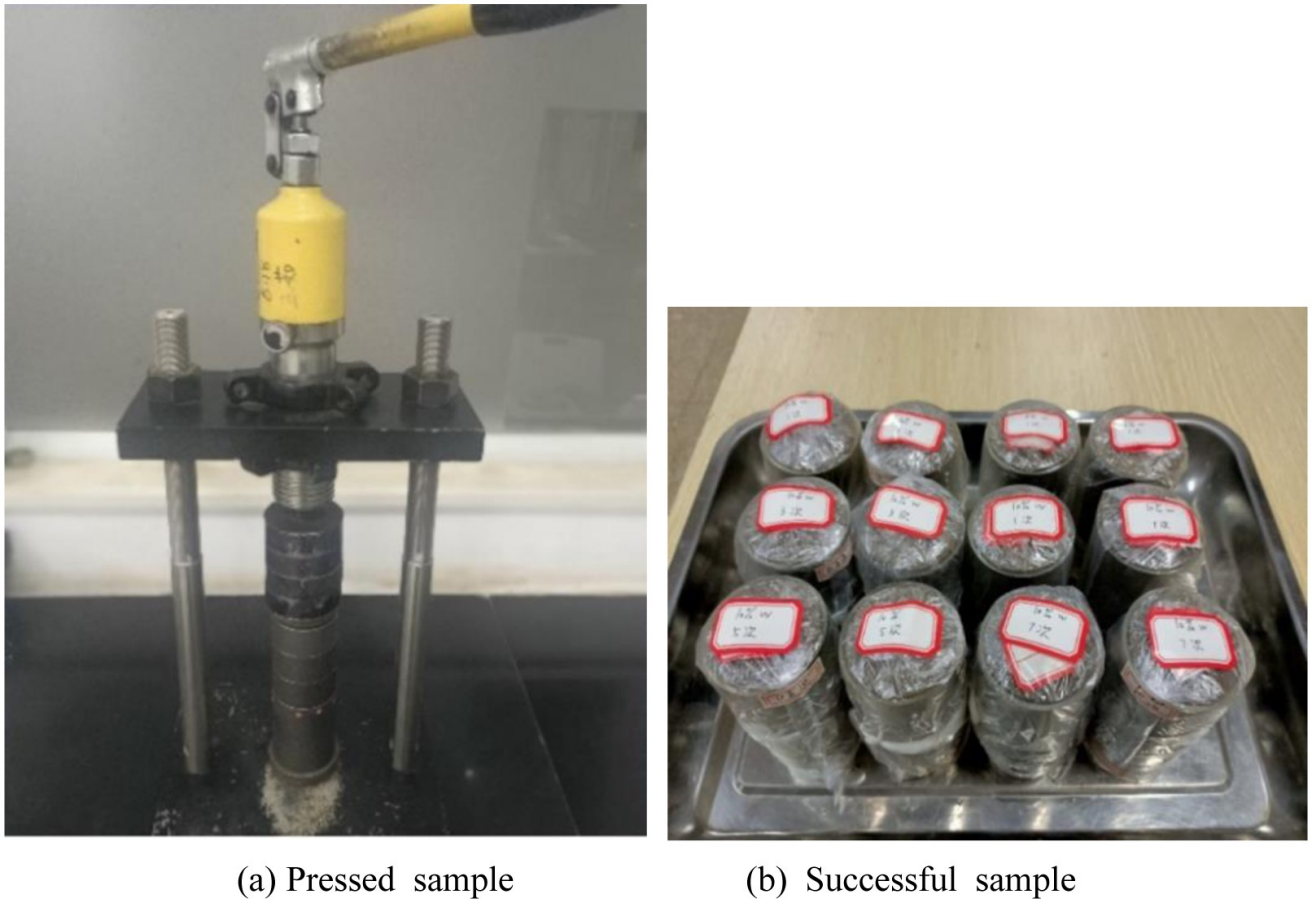
Specimen preparation proced.

The specific test steps are as follows:

1. Screening: method is used to remove particles larger than 5mm from the wet soil mixture. After screening, the soil samples are placed in sealed plastic bags and kept moist for 24 hours.
2. Sample preparation: The wet soil is layered in a mold, forming a cylindrical sample with a diameter of 50mm and a height of 100mm. It is then pressed using the static pressure method.
3. Demoulding and freezing: The sample is quickly frozen in a thermostat set to -30°C for 4 hours to prevent water migration.
4. Maintenance and freeze-thaw cycle: Each freeze-thaw cycle consists of freezing at -10°C for 24 hours, followed by thawing at room temperature (20°C) for 24 hours. The sample is then cured at room temperature (20°C) for an additional 24 hours. The test sample is shown in Fig 1.

China’s highway vehicle speed regulations stipulate that the vibration frequency of the driving load should not exceed 3Hz. In line with this requirement, the vibration frequencies for this experiment have been set at 1Hz, 2Hz, and 3Hz, taking into consideration the findings of previous studies. The specific test conditions are outlined in Table 1:

**Table 1:** Dynamic triaxial test conditions.

Sample number	Freeze-thaw cycles (number)	Moisture content (%)	Confining pressure (kPa)	Dynamic stress amplitude (kPa)	Vibration frequency (Hz)
DR1	0	10	100	0.4 times static strength	2
DR2	1				
DR3	3				
DR4	5				
DR5	7				
HS1	1	6	100	0.4 times static strength	2
HS2		8			
HS3		10			
WY1	1	10	50	0.4 times static strength	2
WY2			100		
WY3			150		
FZ1	1	10	100	0.2 times static strength	2
FZ2				0.4 times static strength	
FZ3				0.6 times static strength	
ZP1	1	10	100	0.4 times static strength	1
ZP2					2
ZP3					3

## 3. Test instruments

**Fig 2:**
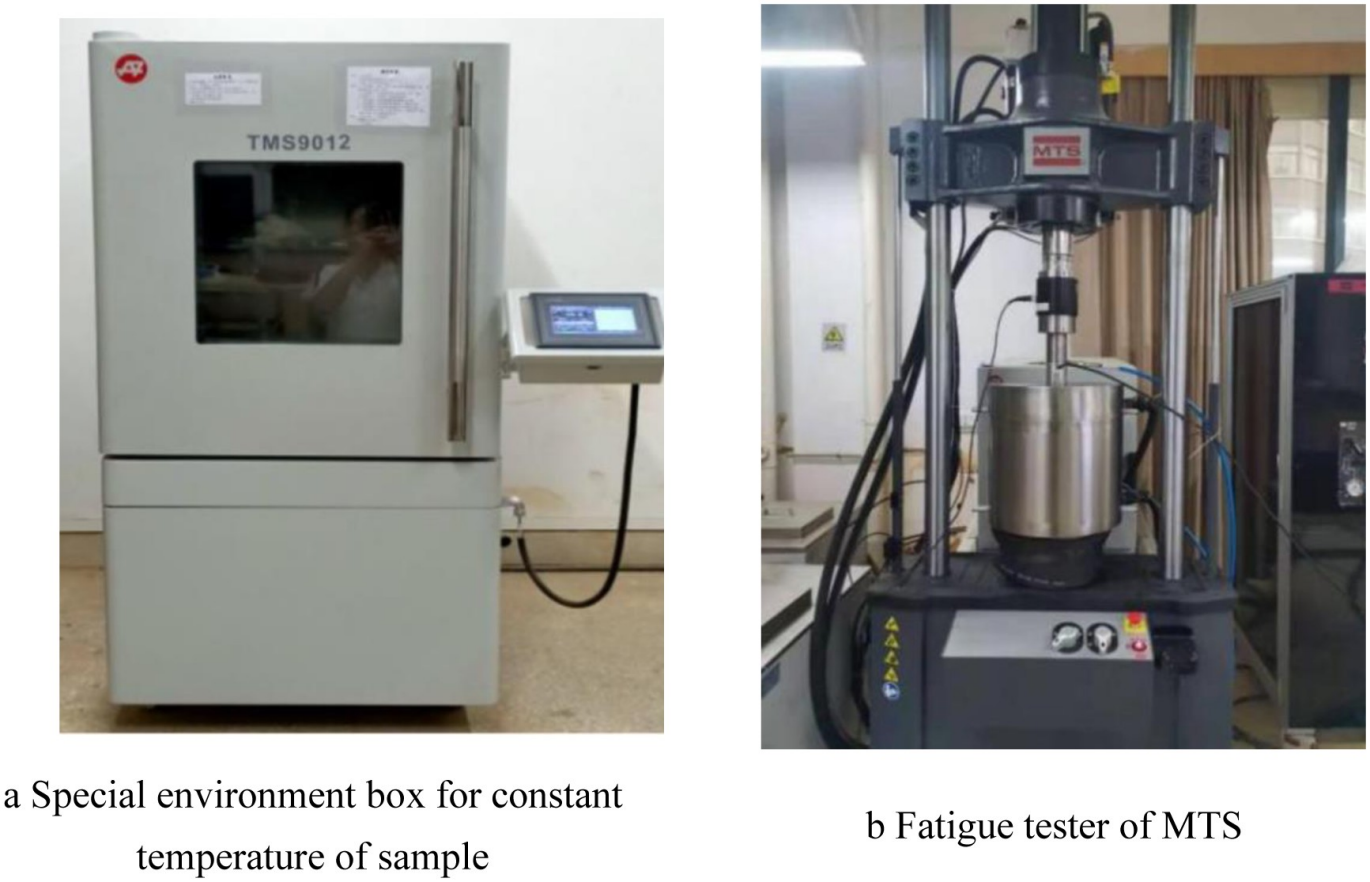
Test apparatus.

The Landmark 370.25 triaxial test machine, manufactured by MTS in the United States, was utilized for this test, as depicted in Fig 2. The equipment’s primary technical specifications are as follows: a maximum axial force of 250 kN, a maximum axial displacement of ± 88 mm, a dynamic load vibration frequency range from 0 to 20 Hz, a temperature range of -40°C to +60°C (with an error of ± 0.1°C), and a measurement accuracy of ± 0.01 to 0.03. The machine can automatically gather data on axial load, axial displacement, confining pressure, time, and other parameters, ensuring precise measurements.

**Fig 3:**
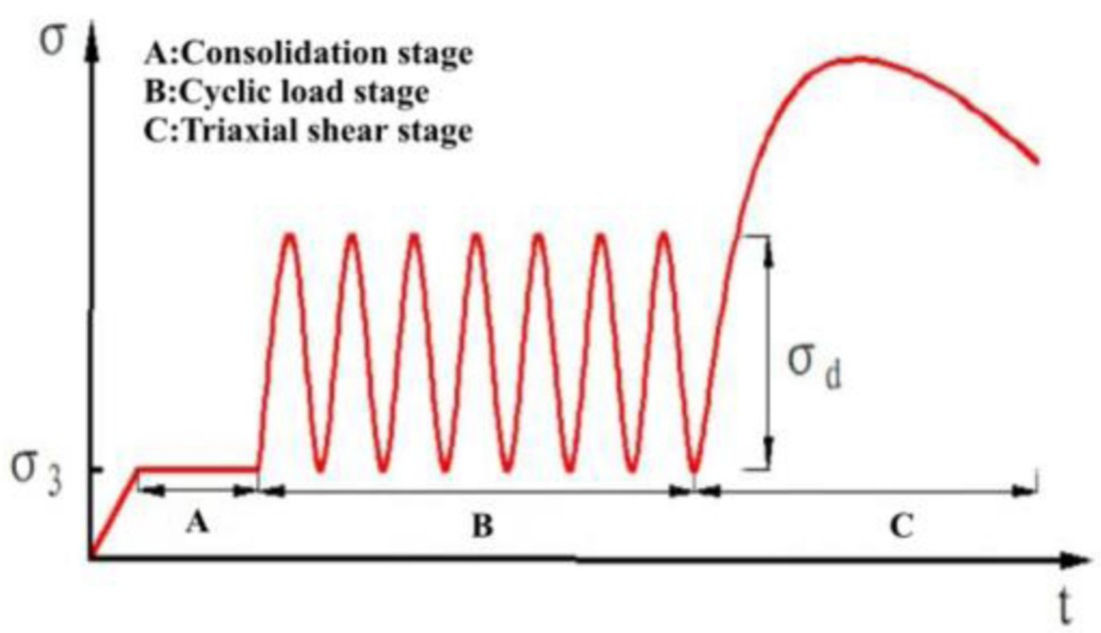
The loading program schematic diagram.

To begin, it is necessary to write the relevant program for the dynamic triaxial test in the control software. Initially, the axial stress is loaded to half of the dynamic stress amplitude, followed by the application of cyclic load in the form of a sine wave. The number of cyclic load vibrations is set at 10,000, and the frequency of data collection is set to 50 Hz. In order to simulate the subgrade’s strength after enduring numerous vehicle loads over an extended period, the sample is not removed after the vibration triaxial test. Instead, a static triaxial shear test is conducted, as shown in Fig 3, to further the analysis.

## 4. Test results and analysis

**Fig 4:**
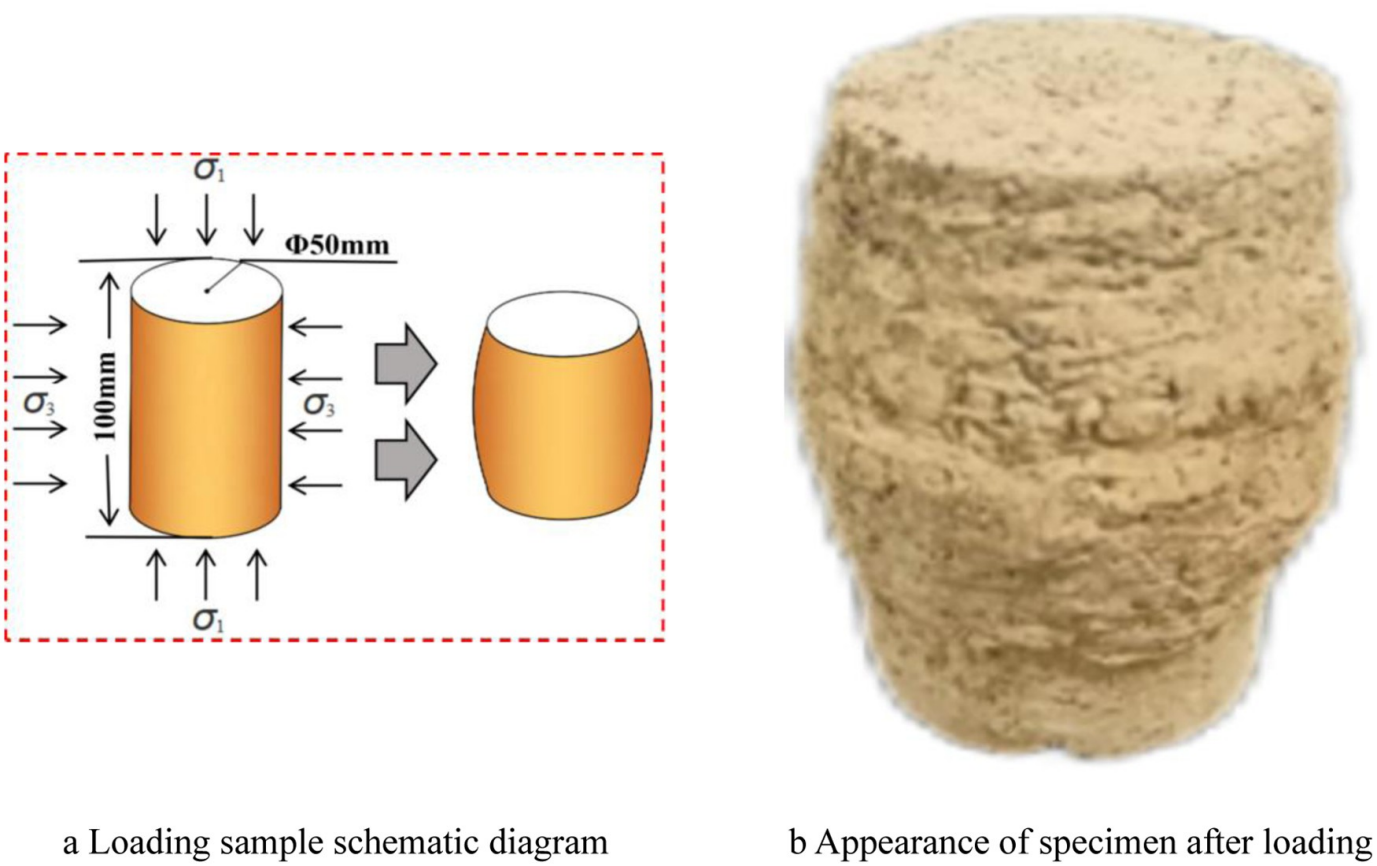
Loading sample schematic diagram and appearance diagram.

The schematic diagram and appearance diagram of the loaded sample are shown in Fig 4. It is evident that the sample, after loading, has taken on a drum-shaped form, indicating dilatancy failure. The smoothness of the side wall of the sample has deteriorated, with the middle portion becoming concave and convex. The middle and lower parts of the sample exhibit noticeable bulging, with the greatest transverse deformation occurring in the middle section, while the top and bottom experience minimal deformation. Overall, the sample has assumed a drum-like shape. The presence of shear bands on the side wall of the sample is not prominent. Cracks can be observed in the upper and middle regions of the sample, with these cracks exhibiting an oblique distribution at an angle greater than 45°. The cracks also display different directions of distribution; some are oriented in one direction while others manifest in two, forming "V" and "∧" types.

### 4.1 Effect of freeze-thaw cycles on deformation characteristics and strength

**Fig 5:**
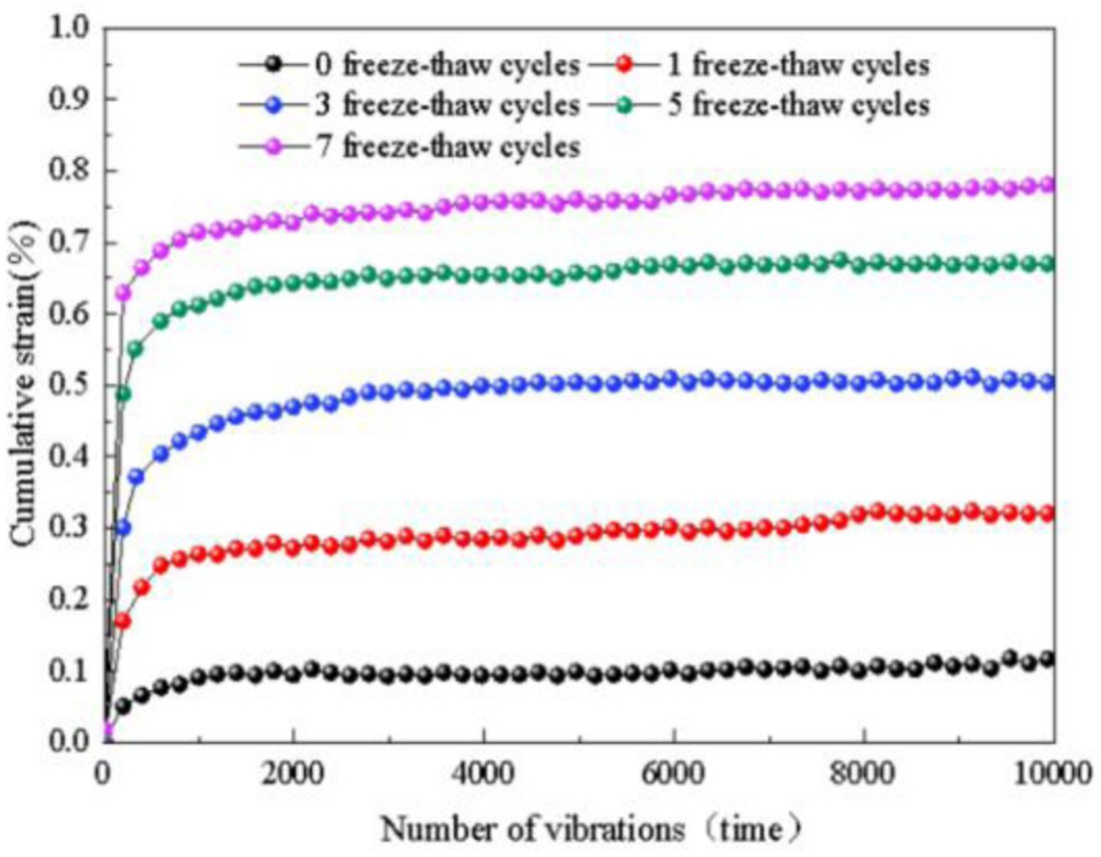
The effect of freeze-thaw cycles on cumulative strain.

The influence of the freeze-thaw cycle coefficient on cumulative strain is determined through experimentation, as illustrated in Fig 5. The diagram reveals that during the initial loading stage (around 500 cycles), the cumulative strain of the subgrade soil increases in an approximately linear fashion, with a faster and larger growth rate, reaching approximately 80% of the final cumulative strain. Subsequently, it enters a nonlinear growth stage where the growth rate gradually decreases. After approximately 5000 cycles, the cumulative strain stabilizes. The final cumulative strain for soil without freeze-thaw cycles at normal temperature is 0.11%, whereas after one freeze-thaw cycle, it increases to 0.32%. After three freeze-thaw cycles, the final cumulative strain is 0.51%, and it further increases to 0.67% after five freeze-thaw cycles. Finally, after seven freeze-thaw cycles, the final cumulative strain reaches 0.78%. As the number of freeze-thaw cycles increases, the final cumulative strain gradually increases. The highest increase is observed after the first freeze-thaw cycle, followed by a slower growth rate. The average growth rate remains below 0.2%, and the final growth rate remains below 1%. This indicates that the subgrade soil is more stable when compacted to a degree of 96%, and exhibits settlement deformation of less than 1% under traffic load.

**Fig 6:**
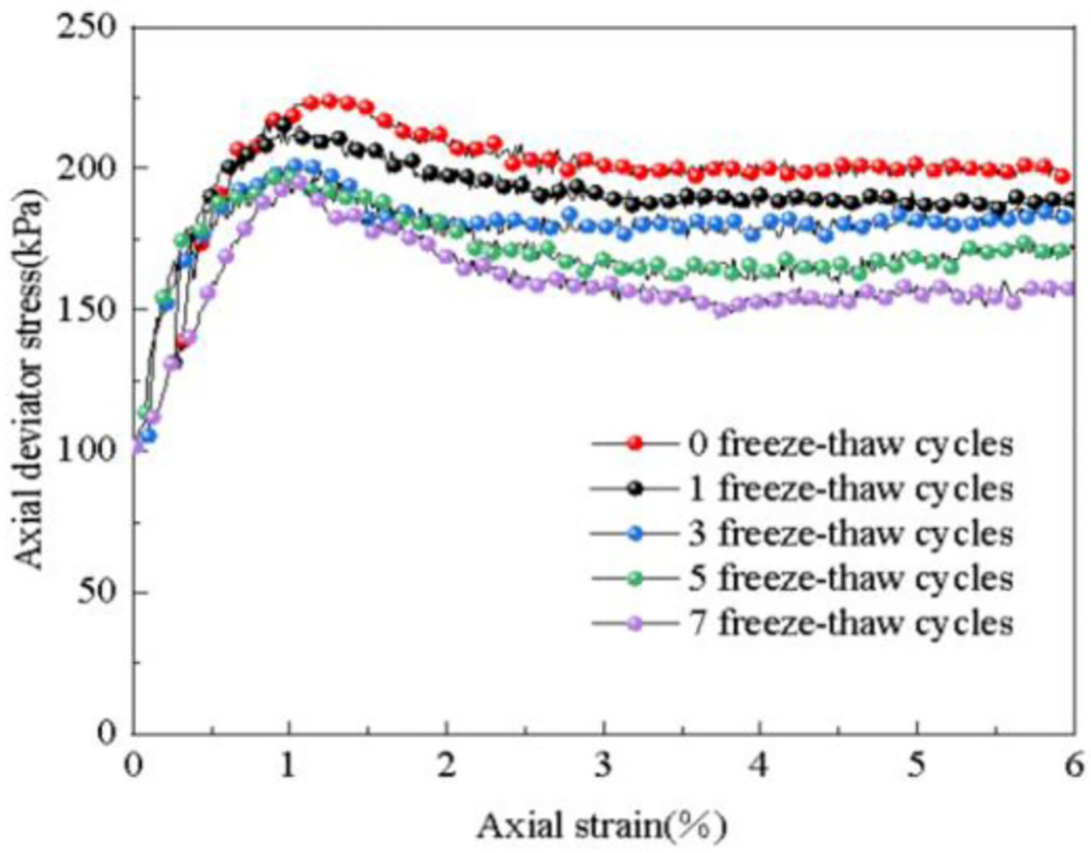
Influence of freeze-thaw cycles on stress—Strain relations.

The influence of the number of freeze-thaw cycles on the stress-strain relationship is determined through experiments, as depicted in Fig 6. The stress-strain curves of the subgrade soil under different freeze-thaw cycles (0, 1, 3, 5, 7 times) after dynamic loading are shown. From the diagram, it is evident that as the number of freeze-thaw cycles increases, the stress-strain relationship curve for the soil samples after dynamic loading follows a similar pattern, all exhibiting softening curves. As the axial strain increases, the axial deviatoric stress initially rises sharply, reaching its peak at 1% axial strain, and then gradually decreases. The rate of decline gradually slows down until it eventually stabilizes. The strength of the subgrade soil noticeably decreases as the number of freeze-thaw cycles increases.

**Fig 7:**
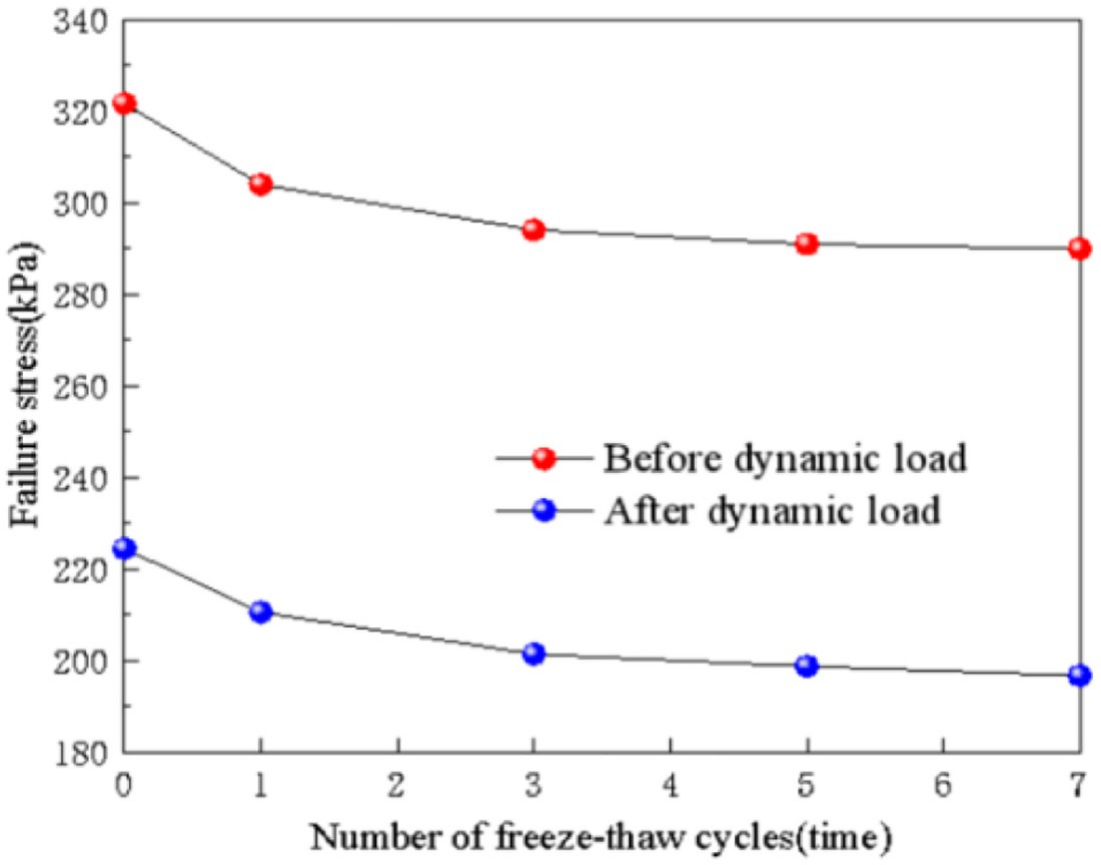
Freeze-thaw cycles—Failure stress relation diagram.

The relationship between the number of freeze-thaw cycles and the failure stress can be determined through experiments, as shown in Fig 7. It is evident from the figure that even after applying dynamic load, the number of freeze-thaw cycles still affects the strength index of the soil. As the number of freeze-thaw cycles increases, the failure stress of the subgrade soil following dynamic load exhibits a roughly exponential decrease. After 7 freeze-thaw cycles, the failure stress of the subgrade soil decreased from 224.52 kPa to 196.76 kPa, representing a decrease of 12.4%. Notably, the most significant decrease in failure stress occurs after the first freeze-thaw cycle, with a reduction of 6.2%. The trend of failure stress of the subgrade soil remains similar before and after applying dynamic load. Under the influence of dynamic load, the failure stress of the subgrade soil noticeably decreases. However, as the number of freeze-thaw cycles increases, the rate of decrease gradually slows down from 30.2% to 28.7%. This indicates that the impact of dynamic load on soil strength diminishes with increasing freeze-thaw cycles, although the decrease is limited and can be considered relatively constant.

During freeze-thaw cycles, the water within the soil undergoes redistribution. With each cycle, the water gradually moves towards the lower part of the soil. As a result, moisture is no longer evenly distributed throughout the soil, but rather becomes layered. This change in moisture distribution alters the contact between soil particles, thereby reducing the friction among them. Consequently, the compressive strength of the soil continuously decreases.

### 4.2 Effect of moisture content on deformation characteristics and strength

**Fig 8:**
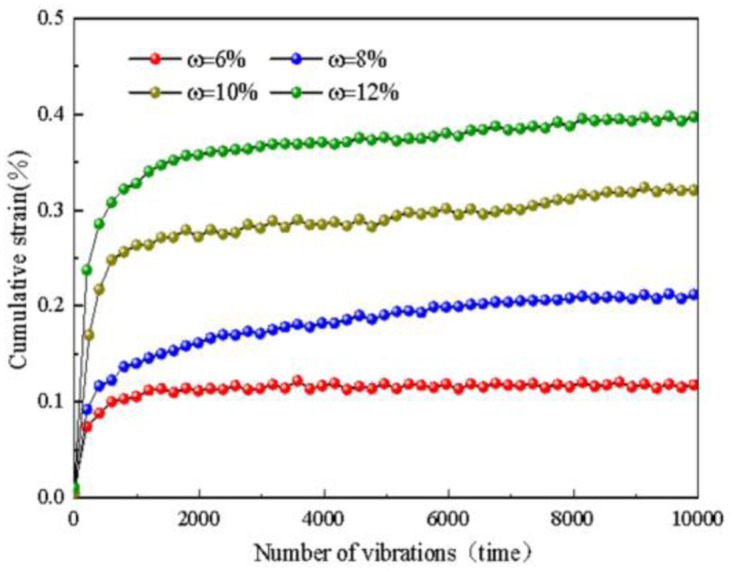
Influence of water content on accumulated strain.

The influence of water content on the cumulative strain of frozen sand under dynamic load was determined through experiments, as shown in Fig 8. The graph illustrates the development of cumulative strain with vibration times under different water content conditions. From the diagram, it can be concluded that during the initial stage of loading (around 500 cycles), the cumulative strain of the subgrade soil increases approximately linearly, with a faster growth rate and larger increment, reaching about 80% of the final cumulative strain. It then enters a nonlinear growth stage, with the growth rate gradually decreasing. After 10,000 cycles, the cumulative strain stabilizes.

For a water content of 6%, the final cumulative strain of the subgrade soil is 0.1175%. For a moisture content of 8%, the final cumulative strain is 0.212%. With a water content of 10%, the final cumulative strain reaches 0.32%. Finally, for a water content of 12%, the final cumulative strain is 0.397%. As the water content increases, the final cumulative strain gradually increases, with an average growth rate of less than 0.1% and a final growth rate of less than 0.3%.

**Fig 9:**
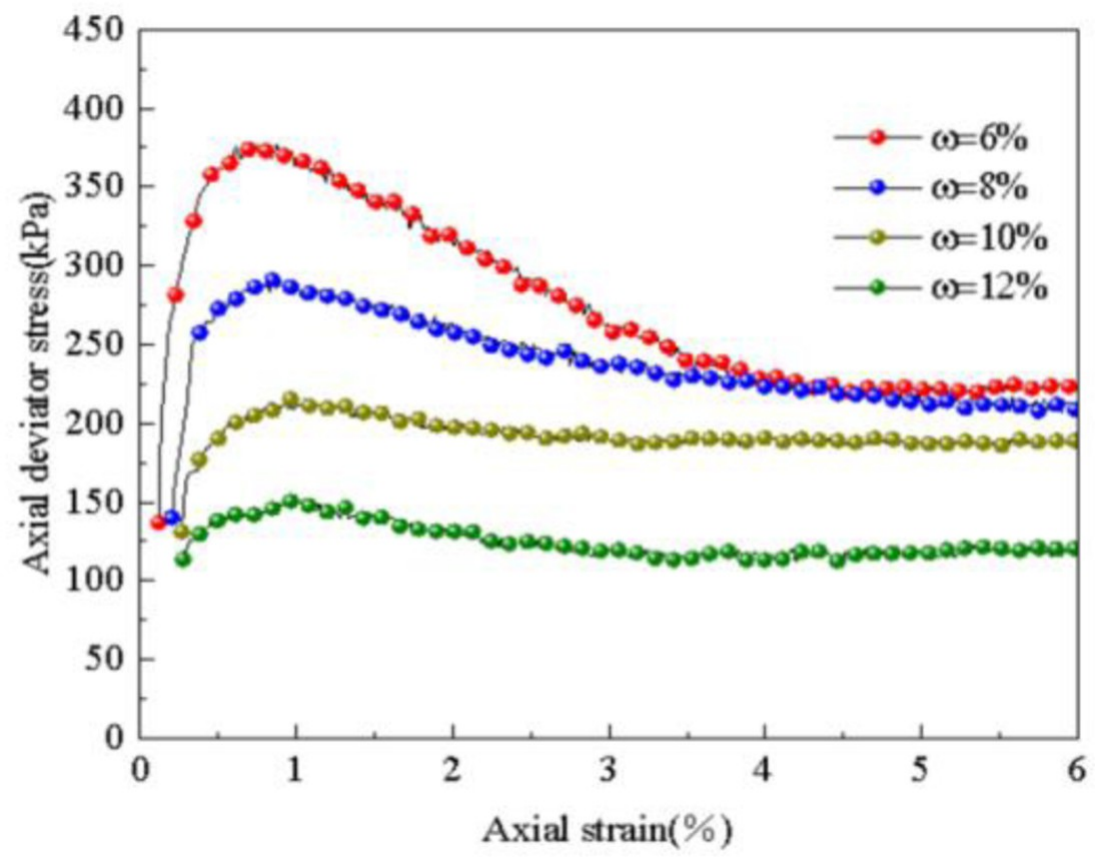
Influence of water content on stress—Strain relations.

Through the stress-strain curve of the subgrade soil under different water content levels (6%, 8%, 10%, 12%), as shown in Fig 9, it is evident that the stress-strain relationship curves of the subgrade soil exhibit softening behavior. The peak point is reached at 1% axial strain. As the water content increases, the axial deviatoric stress decreases. Notably, a lower water content leads to a more pronounced softening phenomenon in the stress-strain curve of the static triaxial test after dynamic load. Consequently, the strength of the subgrade soil noticeably decreases with increasing water content.

**Fig 10:**
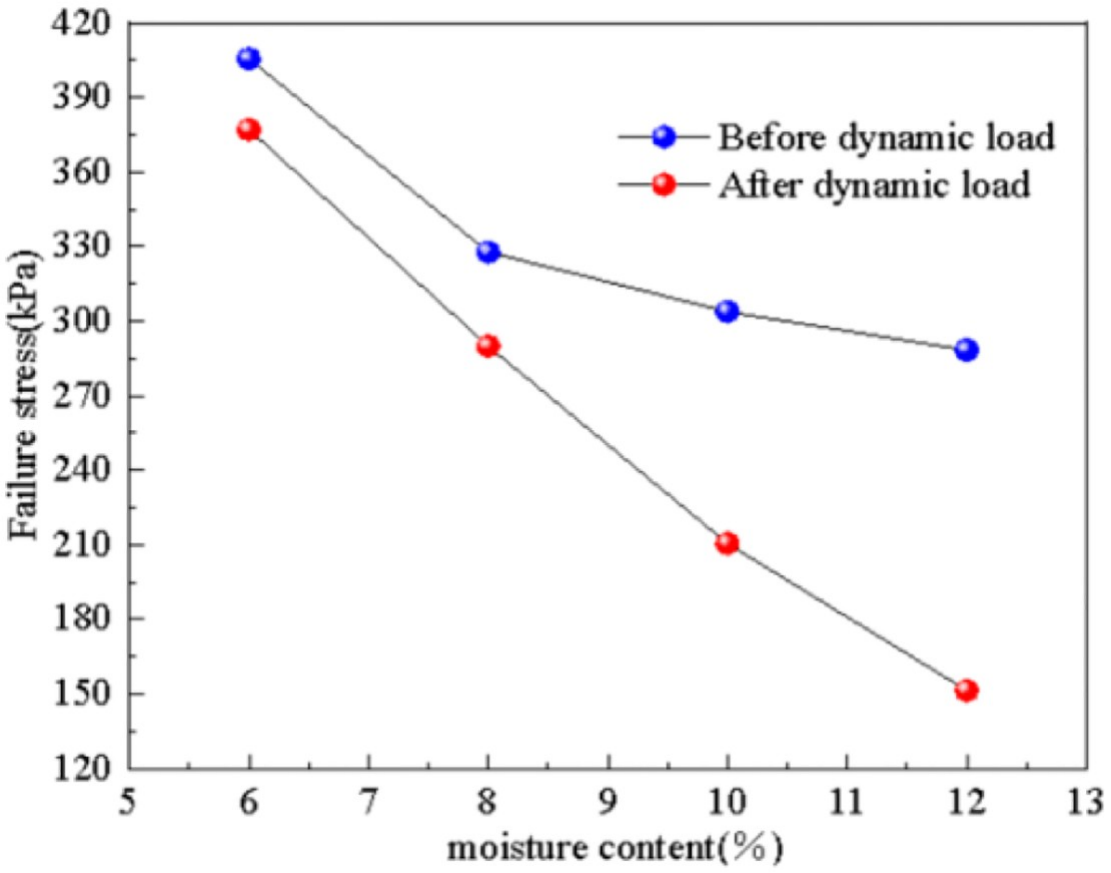
Water content—Failure stress relation diagram.

The influence of water content on the failure stress of subgrade soil, both before and after dynamic loading, was obtained through experimentation, as depicted in Fig 10. It is evident from the figure that even after dynamic loading is applied, water content continues to impact the soil’s strength index. As the water content increases, the failure stress of the subgrade soil after dynamic loading decreases linearly, from 377.1 kPa to 151.5 kPa, representing a decrease of 59.8%. The change in failure stress of the subgrade soil before and after dynamic loading follows a different trend. Under the influence of dynamic loading, the failure stress of the subgrade soil significantly decreases, and as the water content increases, this decrease progressively ranges from 7.0% to 33.7%. This indicates that an increase in water content leads to soil saturation and softening, thereby reducing its ability to withstand dynamic loading. The influence of dynamic loading on soil strength intensifies with higher water content. When the soil is dry and has low water content, the impact of dynamic loading on soil strength is minimal.

When the water content in the soil increases, it fills the tiny pores between soil particles. This leads to lubrication between the particles and reduces friction. Additionally, water adsorbs onto the particle surfaces, creating intermolecular adsorption force that reduces the bonding force between particles. The increase in moisture content causes the distance between soil particles to increase, resulting in a reduction in soil volume. This volume change can alter the arrangement of soil particles and impact the structural stability of the soil. Therefore, as the water content increases, the internal friction and cohesive force of the soil decrease. This ultimately leads to a decrease in soil strength and a deterioration of stability.

### 4.3 Effect of confining pressure on deformation characteristics and strength

**Fig 11:**
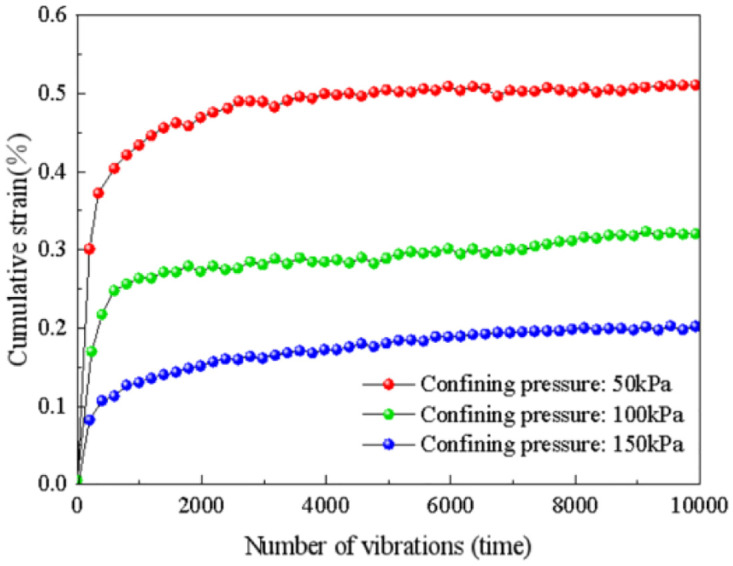
Influence of confining pressure on accumulated strain.

The relationship between cumulative strain and the number of vibrations under various confining pressure conditions is presented in Fig 11. The diagram reveals that the cumulative strain of the subgrade soil increases in a nearly linear fashion within the first 500 cycles, displaying a rapid growth rate. Subsequently, it enters a non-linear growth stage, and the rate of growth gradually decreases. After reaching 10,000 cycles, the cumulative strain stabilizes.

Specifically, the final cumulative strain of the subgrade soil under a confining pressure of 50 kPa is 0.51%, while under a confining pressure of 100 kPa, it is 0.32%. Under a confining pressure of 150 kPa, the final cumulative strain is 0.2%. Remarkably, as the confining pressure increases, the final cumulative strain decreases in an almost linear manner, with an average decrease of less than 0.2% and a maximum decrease of 0.4%. This observation signifies that the increase in confining pressure amplifies the consolidation degree of the soil, making it more challenging for soil particles to flow under dynamic loads. Consequently, the soil’s ability to resist deformation strengthens.

**Fig 12:**
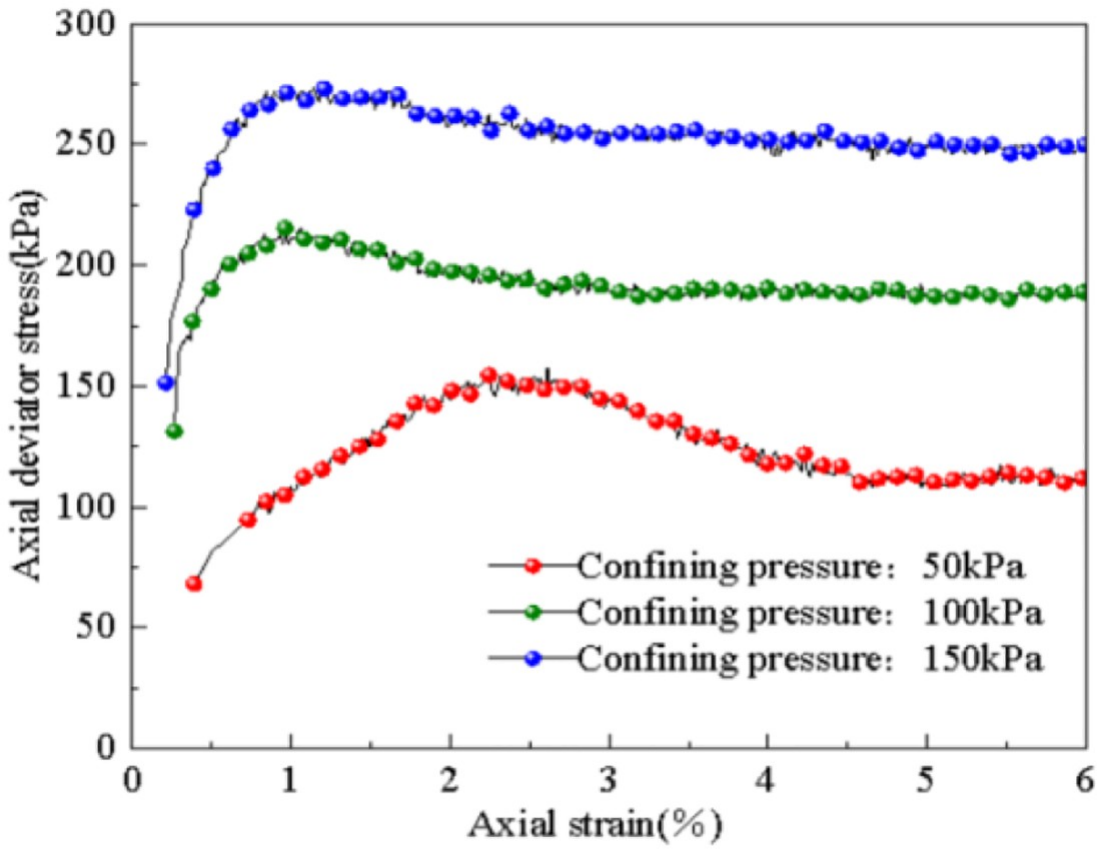
Influence of confining pressure on stress-strain relations.

The stress-strain curves of subgrade soil under different confining pressures (50 kPa, 100 kPa, 150 kPa) are shown in Fig 12. From the diagram, it is evident that the stress-strain curve of the subgrade soil softens as the confining pressure increases. The softening phenomenon of the subgrade soil after dynamic load becomes more pronounced as the confining pressure decreases. Moreover, as the confining pressure increases, the axial strain needed to reach the peak point of deviatoric stress decreases. Consequently, the strength of the subgrade soil significantly increases with higher confining pressures. This illustrates that, even after the application of dynamic load, the confining pressure continues to impact the soil’s strength. Despite the original skeleton structure of the subgrade soil being disrupted under dynamic load, the increase in confining pressure leads to soil compaction and reduced pore space, resulting in the formation of a new stability system and an improved strength of the subgrade soil.

**Fig 13:**
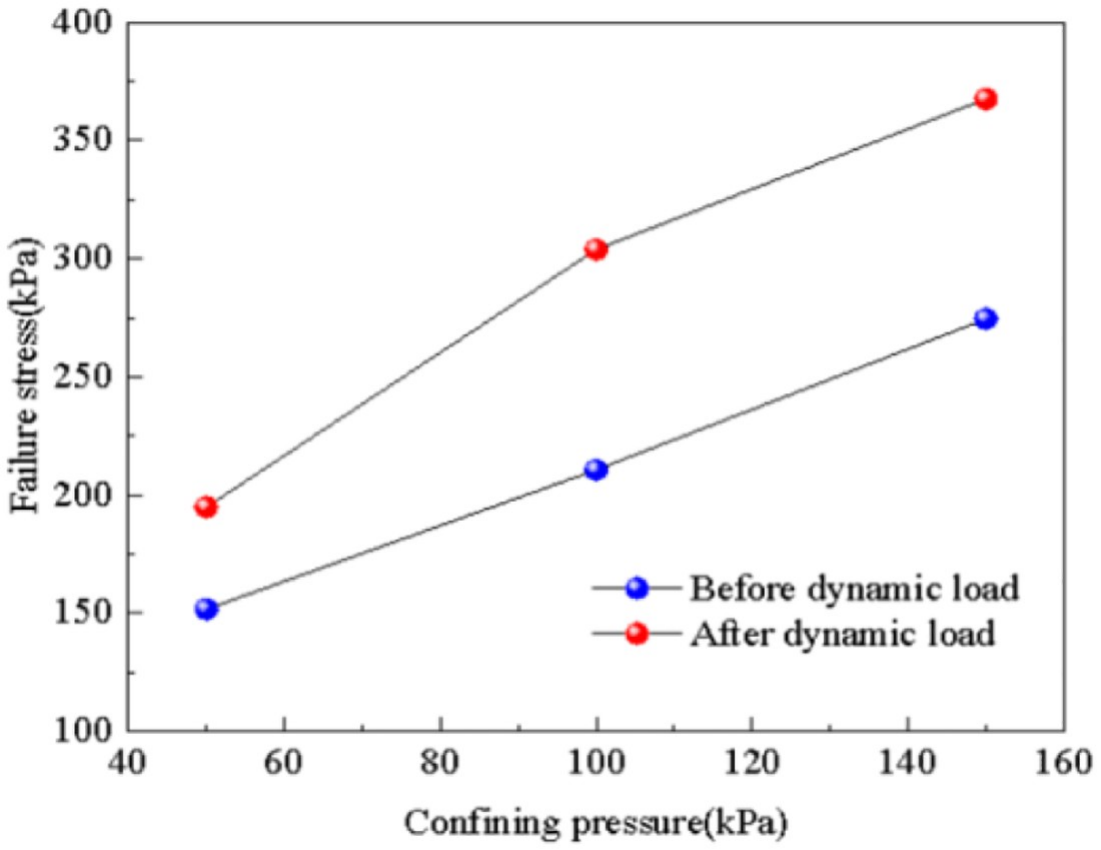
Confining pressure—Failure stress relation diagram.

The influence of confining pressure on the failure stress of subgrade soil before and after dynamic loading is determined through experimentation, as shown in Fig 13. It is evident from the figure that even after the application of dynamic loading, the strength index of the soil is still affected by the confining pressure. The failure stress of the subgrade soil increases approximately linearly with the increase in confining pressure, from 151.6 kPa to 274.5 kPa, resulting in an 81.1% increase. The change in trend of the failure stress of the subgrade soil before and after dynamic loading is similar. Under the influence of dynamic loading, the failure stress of the subgrade soil decreases. As the confining pressure increases, the magnitude of the decrease ranges from 22.2% to 47.8%, indicating that the influence of dynamic loading on strength intensifies with increasing confining pressure.

**Fig 14:**
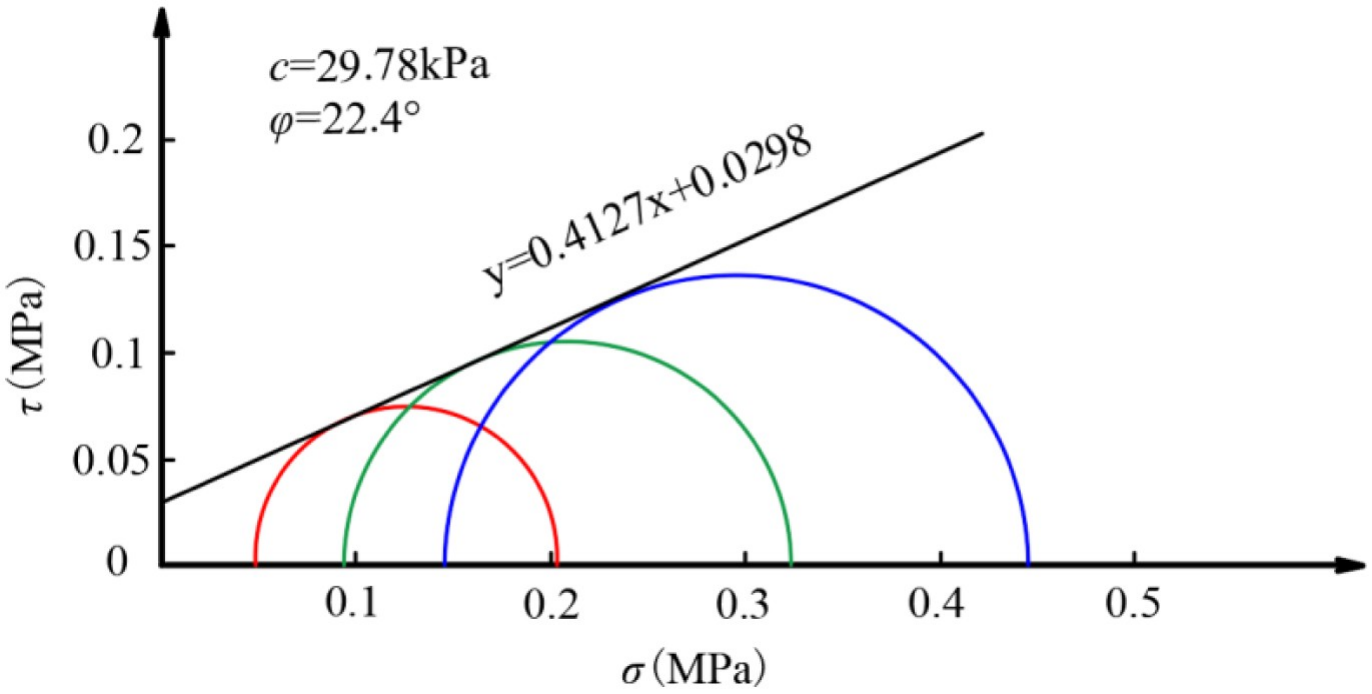
The molar envelope.

According to the test results, a Mohr stress circle (also known as the ultimate stress circle) is constructed for the subgrade soil under various confining pressures. Fig 14 depicts this circle along with its common tangent. The formula for the common tangent is derived. Through calculations, the cohesion of the subgrade soil after experiencing dynamic load is determined to be 29.78 kPa, while the internal friction angle measures 22.4°. A comparison with the results obtained prior to the application of dynamic load reveals a decrease in cohesion by 5.8 kPa and a decrease in the internal friction angle by 5°. These findings indicate that the original structure of the subgrade soil is compromised upon the application of dynamic load, resulting in a reduction in strength due to relative particle displacement.

Under low confining pressure, the primary effect is the compression of pore volume, without any fundamental changes to the microstructure of the soil. As the confining pressure increases, it not only compresses the pore volume, but also results in the closure of soil macropores and the collapse of the overhead structure. These changes alter the microstructure of the soil, causing the soil particles to become more compact and dense, and the soil skeleton to become harder. This structural transformation enhances the soil’s ability to withstand external deformation, thereby increasing its shear strength.

### 4.4 Effect of loading rate on strength

**Fig 15:**
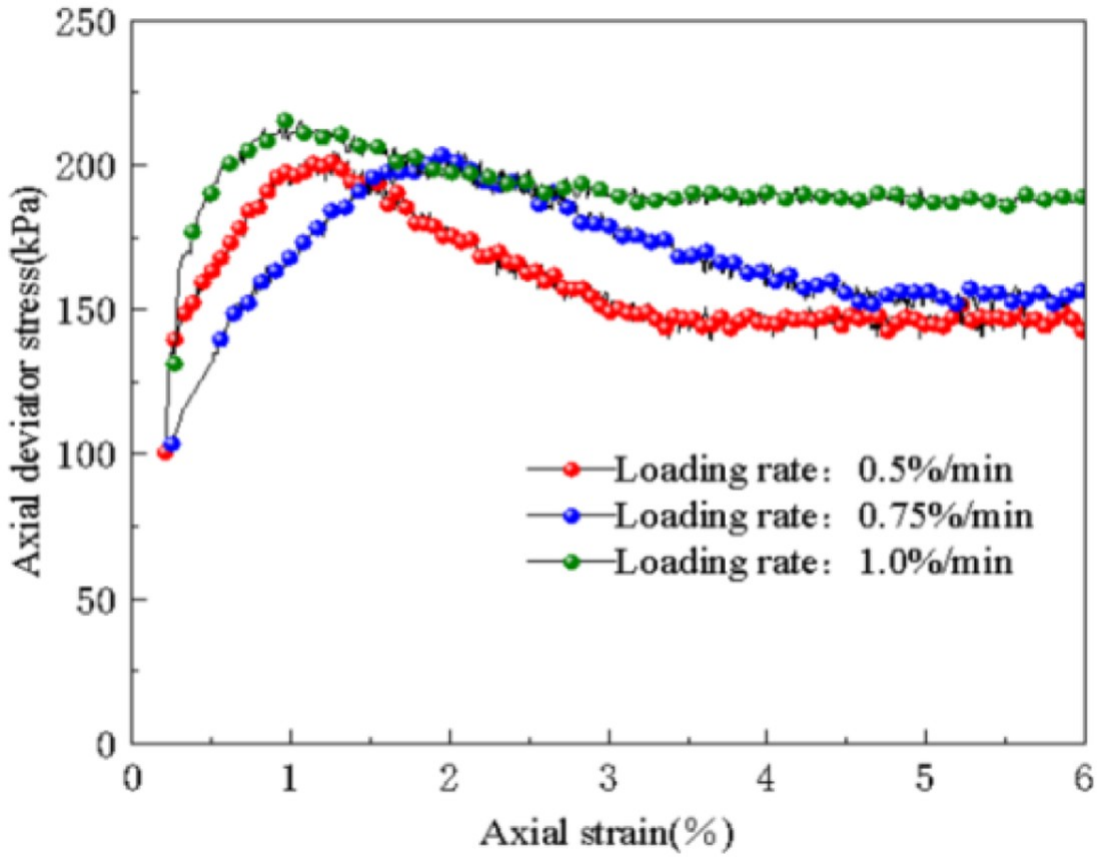
Influence of loading rate on stress-strain relations.

The stress-strain curves of the subgrade soil under different loading rates (0.5% / min, 0.75% / min, 1% / min) are depicted in Fig 15. It is evident from the figure that as the loading rate increases, the stress-strain relationship curves of the subgrade soil exhibit a softening trend, and the curve shape is irregular. The increase in axial deviatoric stress is minimal, indicating a weak impact of the loading rate.

**Fig 16:**
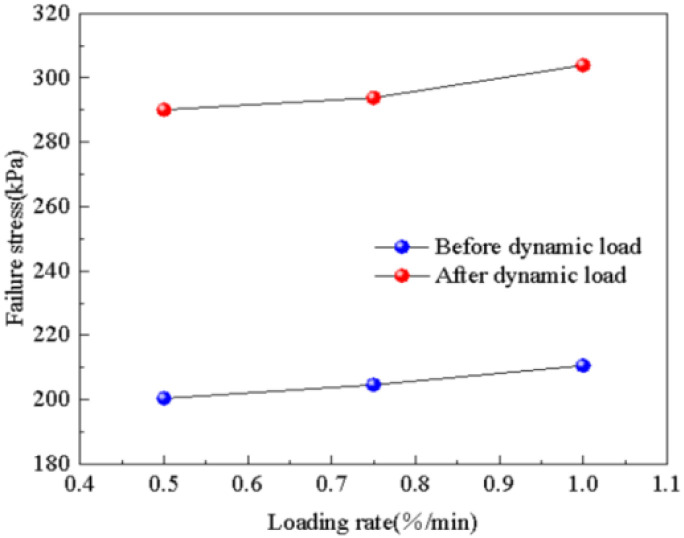
Loading rate—Failure stress relation diagram.

The influence of loading rate on the failure stress of subgrade soil before and after dynamic load is shown in Fig 16. It can be observed from the diagram that the failure stress of subgrade soil after dynamic load increases in a nearly linear manner with the increase in loading rate, rising from 200.46 kPa to 210.62 kPa, which represents a 5.1% increase. The change in the failure stress of subgrade soil before and after dynamic load exhibits a similar trend. Under the effects of dynamic load, the failure stress of subgrade soil decreases, with a comparable magnitude of reduction.

Soil is a material that exists in a three-phase equilibrium of solid, liquid, and gas. When the loading speed is high, the water and air in the soil may not be able to escape easily. As closed water and air are hard to compress, a temporary resistance occurs, giving the illusion of increased soil strength during the loading process. However, this is not a genuine increase in strength, but rather a superficial effect that occurs only during the loading process.

## 5. Discussion

Many scholars have extensively researched the mechanics and deformation mechanisms of highway subgrades in cold regions. They have utilized advanced testing techniques and instruments to measure the comprehensive effects of temperature, water content, freeze-thaw cycles, and other factors on the mechanical properties of subgrade soil in cold regions. However, these studies have mainly focused on either static load or dynamic load, with limited research on subgrade soil under the combined effect of dynamic and static loads. This paper aims to discuss the influence of freeze-thaw cycles, water content, confining pressure, and loading rate on subgrade deformation under vehicle load. Additionally, it proposes an innovative simulation of the subgrade passing through the vehicle and studies its mechanical properties, drawing important conclusions. In cold regions, the freeze-thaw cycle, in particular, has a significant impact on subgrade performance. This is because the freeze-thaw cycle causes the redistribution of water within the soil and alters the contact between soil particles. However, it is important to note that this study has certain limitations. For instance, future research will explore the impact of intermittent vehicle load and the influence of different materials on subgrade performance.

## 6. Conclusion

In this paper, we focus on the silty subgrade soil in Northwest China. We aim to investigate the impact of various factors on the deformation characteristics and mechanical properties of subgrade soil under cyclic loading. And the influence of long-term vehicle load on subgrade strength is considered. The following conclusions have been drawn from our experiments:

1. The law of the cumulative strain curve of subgrade soil can be described as rapid linear growth in the early stage, followed by slow nonlinear growth in the middle stage, and finally reaching a stable state in the later stage. The cumulative strain shows a positive correlation with the number of freeze-thaw cycles, resulting in a final cumulative strain of 0.78%. Additionally, the cumulative strain is positively correlated with the water content, leading to a final cumulative strain of 0.397%. On the other hand, the cumulative strain exhibits a negative correlation with the confining pressure, resulting in a final cumulative strain energy of 0.2%.
2. As the number of freeze-thaw cycles increases, the failure stress of subgrade soil under dynamic load decreases from 224.52 kPa to 196.76 kPa, following an approximately exponential decreasing trend. The influence of dynamic load on soil strength diminishes with an increasing number of freeze-thaw cycles.
3. As the water content increases, the failure stress of subgrade soil decreases from 377.1 kPa to 151.5 kPa under dynamic load. In this case, the influence of dynamic load on soil strength intensifies with an increasing water content.
4. As the confining pressure increases, the required axial strain to reach the peak deviatoric stress decreases, and the failure stress increases from 151.6 kPa to 274.5 kPa. The strength of the subgrade soil significantly improves with higher confining pressure. The failure stress of the subgrade soil after dynamic loading increases in a nearly linear manner with increasing confining pressure, and the effect of dynamic load on strength also increases with higher confining pressure.
5. As the loading rate increases, the stress-strain curves of the subgrade soil exhibit a softening trend, and the failure stress of the subgrade soil increases from 200.46 kPa to 210.62 kPa. The failure stress of the subgrade soil after dynamic loading also increases in an approximately linear manner with increasing loading rate.
6. Dynamic loading has a softening effect on the stress-strain curve of the subgrade soil. Under dynamic loading conditions, the failure stress of the subgrade soil noticeably decreases.

## Supporting information

S1 Table (XLSX)
